# The genetic scenario of Mercheros: an under-represented group within the Iberian Peninsula

**DOI:** 10.1186/s12864-021-08203-y

**Published:** 2021-12-15

**Authors:** André Flores-Bello, Neus Font-Porterias, Julen Aizpurua-Iraola, Sara Duarri-Redondo, David Comas

**Affiliations:** Departament de Ciències de la Salut i de la Vida, Institut de Biologia Evolutiva (CSIC-UPF), Universitat Pompeu Fabra, 08003 Barcelona, Spain

**Keywords:** Merchero population, Haplotype-based methods, Genome-wide autosomal data, Genetic origin, Minority ethnic group, Iberian Peninsula, Uniparental markers

## Abstract

**Background:**

The general picture of human genetic variation has been vastly depicted in the last years, yet many populations remain broadly understudied. In this work, we analyze for the first time the Merchero population, a Spanish minority ethnic group that has been scarcely studied and historically persecuted. Mercheros have been roughly characterised by an itinerant history, common traditional occupations, and the usage of their own language.

**Results:**

Here, we examine the demographic history and genetic scenario of Mercheros, by using genome-wide array data, whole mitochondrial sequences, and Y chromosome STR markers from 25 individuals. These samples have been complemented with a wide-range of present-day populations from Western Eurasia and North Africa. Our results show that the genetic diversity of Mercheros is explained within the context of the Iberian Peninsula, evidencing a modest signal of Roma admixture. In addition, Mercheros present low genetic isolation and intrapopulation heterogeneity.

**Conclusions:**

This study represents the first genetic characterisation of the Merchero population, depicting their fine-scale ancestry components and genetic scenario within the Iberian Peninsula. Since ethnicity is not only influenced by genetic ancestry but also cultural factors, other studies from multiple disciplines are needed to further explore the Merchero population. As with Mercheros, there is a considerable gap of underrepresented populations and ethnic groups in publicly available genetic data. Thus, we encourage the consideration of more ethnically diverse population panels in human genetic studies, as an attempt to improve the representation of human populations and better reconstruct their fine-scale history.

**Supplementary Information:**

The online version contains supplementary material available at 10.1186/s12864-021-08203-y.

## Background

Genetic studies focused on human populations have been intensely boosted in the last decades owing to the increasingly affordable genome-wide platforms, expanding the amount of high-quality genetic material considered in the studies, and allowing their availability in public genomic repositories. This has enabled to vastly depict the human genetic landscape both from an anthropological and biomedical point of view [1–3]. Yet, many human groups have been systematically underrepresented in such studies, leading to an incomplete picture of the human genetic diversity. Recently, new efforts to include minority ethnic groups represent a step forward to reduce these disparities and towards the understanding of the fine-scale human population history [4, 5].

The Iberian Peninsula genetic scenario is the complex result of several demographic processes related to different historical events characterized by diverse population contacts, including multiple ethnic and geographic origins [6, 7]. The early history of the Iberian Peninsula was characterized by the presence of the Celtiberians, Iberians, Lusitanians, and Tartessians, together with a high influence of Mediterranean cultures, such as Phoenicians, Greeks, and especially the Roman Empire [8]. This was followed by the settlement of the Germanic tribes, before the Iberian Islamic periods which were characterized by a relevant contact with North African populations [9, 10]. These processes led not only to a genetic conglomerate, but also to wealthy cultural and linguistic influences that shape the present Iberian population. The Iberian Peninsula population has been analysed in many genetic studies, showing an internal heterogeneity with genetic geographic patterns following an east-to-west axis [6]. In addition, several population groups have stood out from this general context due to their particular demographic histories and genetic landscapes, as it is the case of Basque [11], Eivissa [12], and Spanish Roma populations [13, 14].

Despite the exhaustive genetic coverage of the Iberian Peninsula, being also part of the 1000 genomes project [1], some population groups have been neglected, which precludes depicting and understanding the demographic history of the entire region. One of these underrepresented groups is the Merchero population, a minority ethnic group that has been historically persecuted and socially invisibilized. They have an itinerant history, share common traditional occupations and speak *quinqui*, an unclassified language. They are nowadays distributed across the whole Iberian Peninsula, although the total number of Mercheros has not been properly estimated [15, 16]. Little is known about the origins of the Mercheros, although several untested hypotheses have been proposed. It has been suggested that Mercheros and Iberian Roma besides having a common nomadic history and cultural characteristics, share the same origin in South Asia. Other literature points to a Moorish origin, due to the presence of Muslim groups in the Iberian Peninsula from the 8th to 15th centuries. Some written records, on the other hand, mention that their origin might be found in other European nomadic groups [15, 17, 18]. Finally, Mercheros could also be the descendants of multiple non-related groups of Spanish land workers that abandoned the feudalism system during medieval times [15, 17, 18]. However, none of these contrasting hypotheses have been formally tested and a genetic approach would provide a reference landmark to the characterization of this underrepresented population group.

In the present study, we aim to genetically characterize the Merchero population in order to complete the genetic landscape of Iberian Peninsula, by analysing genome-wide autosomal and uniparental data from 25 samples. These are the first available Merchero genetic samples, which will reduce the gap of the underrepresented populations and ethnic groups in the reference panels. In order to assess the genetic origin of the Merchero population, their ancestry profiles and admixture levels with external groups are examined with allele-frequency and haplotype-based methods. In addition, we describe their demographic history and genetic substructure, through the inference of runs of homozygosity (ROHs), identity by descent (IBD) segments, and effective population size (Ne) dynamics.

## Results

### Mercheros fall within the genetic context of the Iberian Peninsula with modest evidence of Roma admixture

**Figure 1:**
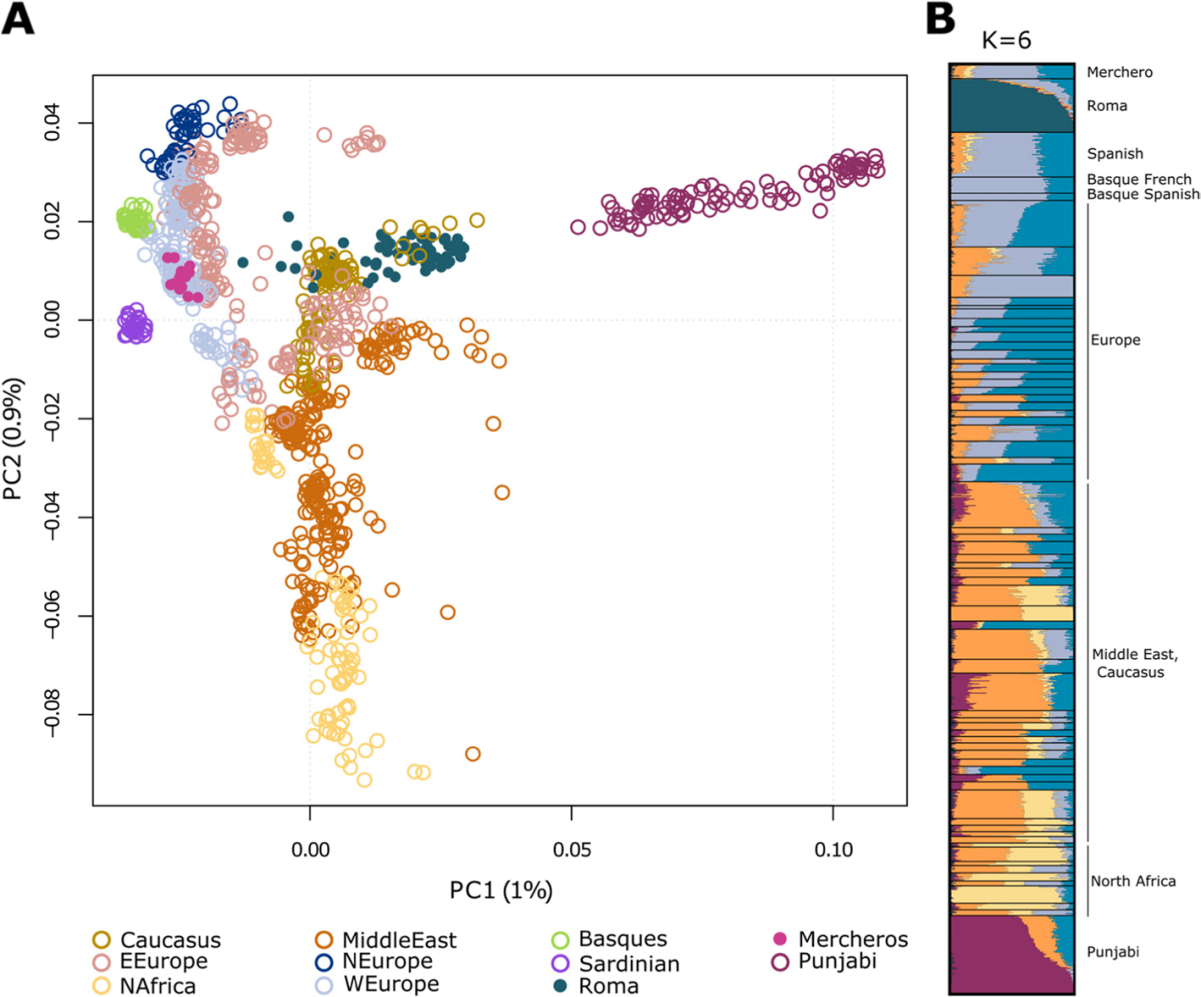
Genetic contextualization of Mercheros. Principal Component **(A)** and Admixture **(B)** analyses in a wide genetic context including the whole dataset are shown. Only K = 6, with the lowest cross-validation error, is shown for the ADMIXTURE analysis. Ks from 2 to 5 and the complete list of populations are presented in Fig. S2

To assess the genetic origin of Mercheros, a large genome-wide database including West Eurasian and South Asian populations individuals was analyzed (see Material and Methods). We first computed a Principal component analysis (PCA) (Fig. 1A), where Merchero samples cluster together with other Spanish individuals, suggesting that they share a similar genetic profile. In the UMAP analysis (Fig. S1), individuals establish stronger local clustering within discrete populations, while Merchero individuals also overlap with the Spanish genetic samples.

The global ancestry of the Merchero population was examined with an ADMIXTURE analysis (Fig. 1B, Fig. S2). At K = 6 (lowest cross-validation error), Mercheros and the rest of Spanish individuals share similar ancestry proportions, with the exception of the Roma-related component (in pink), which is significantly higher in Mercheros (2.380% ± 2.373 and 0.826% ± 1.048, respectively, Wilcoxon test *p*-value = 0.0018). The higher Roma proportion together with a large standard deviation of this component in the Merchero’s groups suggest that few Merchero individuals might have experienced gene flow from Spanish Roma. This result is also supported by the UMAP analysis of the Iberian Peninsula context, where some Merchero samples are placed between the Roma and the rest of the Spanish samples (Fig. S3).

**Fig. 2:**
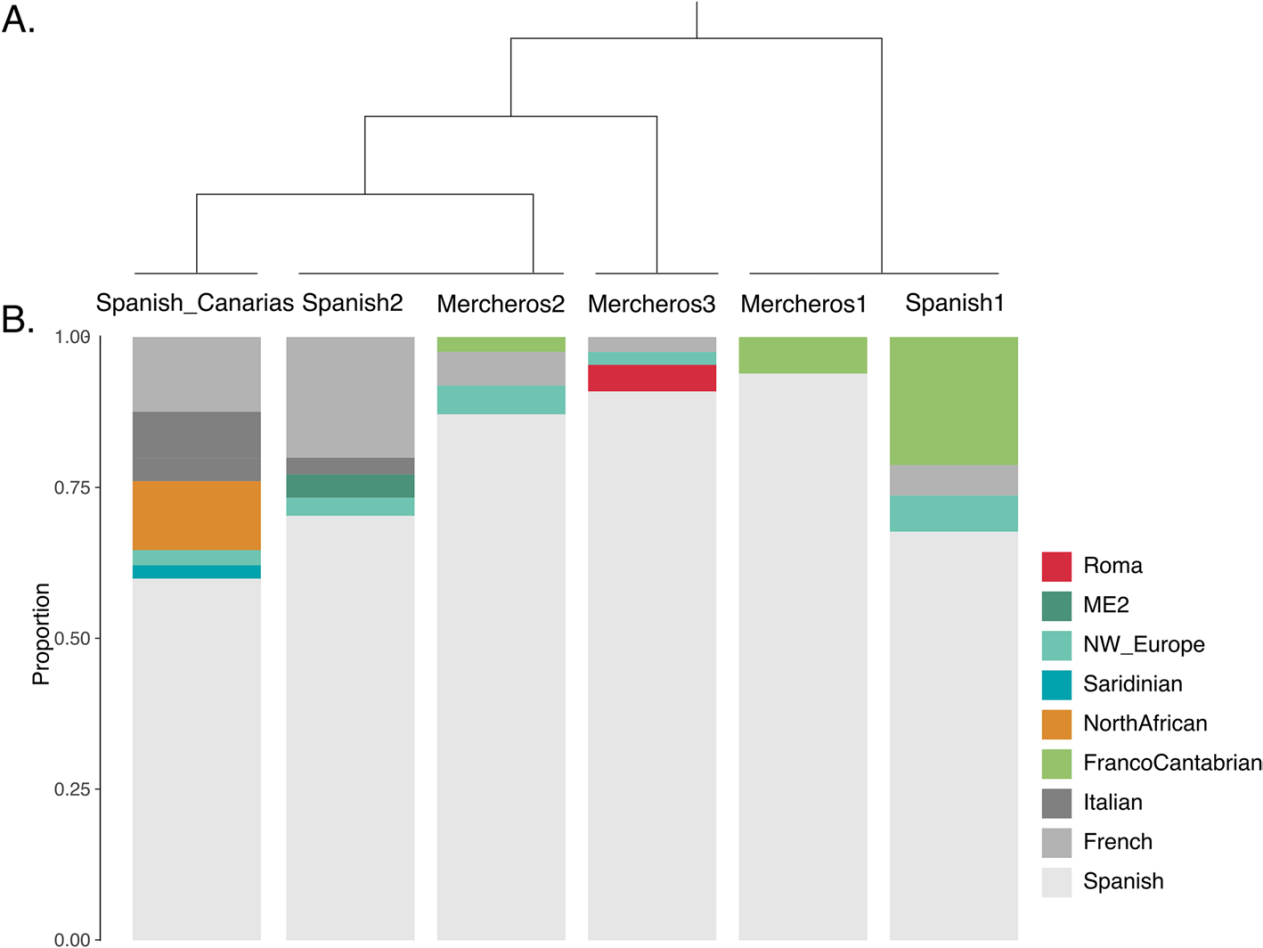
Intrapopulation genetic structure and ancestry components. **A**. Spanish branch of the global fineSTRUCTURE dendrogram. The complete dendrogram is shown in Fig. S4. **B**. Ancestry profile proportions inferred from the NNLS analysis

We further explored the Merchero’s genetic composition using the fine-scale haplotype-based methods ChromoPainter and fineSTRUCTURE. As previously shown in the PCA and UMAP analyses, Mercheros are genetically close to other Spanish samples and they consequently cluster in the same branch of the fineSTRUCTURE dendrogram (Fig. 2A, Fig. S4 and Table S2). However, moderate genetic substructure is observed within this group, where three genetically homogeneous clusters can be differentiated: Merchero1 in the same branch as Spanish1, Merchero2 clustering with Spanish2, and Merchero3 as an independent branch (Fig. 2A). The ancestry profiles of these clusters were obtained from the ChromoPainter coancestry matrix with the “non-negative least squares” (NNLS) method. Both Merchero1 and Merchero2 groups show a similar ancestry composition as Spanish1 and Spanish2, respectively (Fig. 2B). However, Merchero3 profile reveals a non-negligible contribution of Roma-related ancestry (~ 4.5%), confirming the presence of Roma gene flow in some Merchero samples (Fig. 2B). In fact, an AMOVA analysis shows the higher heterogeneity of the Merchero3 group (Table S3). In order to test for an admixture event, GLOBETROTTER was applied to the Merchero3 cluster; however, the result provides an *unclear signal*, and no estimates of an admixture event were available.

The uniparental lineages identified in the Merchero samples are similar to those found in other European populations [19, 20], without distinctive influence from South Asian or North African contributions. The most common mitochondrial haplogroup is H, (present in almost 40% of the Merchero sequences), with H1 being the most common H sublineage. For the Ychr, R1b haplogroup is the most frequent lineage within the dataset (up to 61.5%), consistent with previous observations of uniparental markers in Europe and the Iberian Peninsula [19, 20] (Table S1, S4-S5, Fig. S5).

These results show that Mercheros present a genetic ancestry profile similar to the general Spanish population. A modest and uneven Roma gene flow into Mercheros is detected, suggesting that the genetic admixture between both groups is subtle and not a widespread and inherent characteristic of these populations.

### Mercheros show limited genetic isolation coupled with intrapopulation heterogeneity

In order to describe the population-specific genetic patterns of Mercheros and assess their demographic history, ROHs and IBD segments were explored.

High ROHs values of total number and length (> 4 Mb) are observed in Mercheros (Fig. S6), which are larger than those previously observed in Roma [13, 21]. This suggests signals of recent inbreeding in the Merchero population. However, it is noteworthy that few samples (18%) are responsible for these high ROHs values as shown by the standard deviations and the proportion of individuals represented in the longest ROH categories. In fact, when dissecting the ROH values by the genetic clusters inferred from fineSTRUCTURE, Merchero3 shows the highest, but not statistically significant, median ROH length, immediately below Roma and Basques, whereas Merchero1 and Merchero2 clusters show values close to Spanish and CEU (Table 1, Table S6). Nevertheless, the median cumulative IBD length within groups are similar in all Merchero clusters (without significant differences), and close to Spanish and CEU values (Table 1, Table S6). Therefore, these results show that the observed signals of recent inbreeding come from sample-specific values within the Merchero3 cluster. However, this result should be taken with caution due to the low sample size of Merchero3 cluster.

**Table 1:** Intrapopulation genetic patterns. Sum of ROH length in Mb and IBD in cM for each population cluster. Only ROHs > 1 Mb and IBD segments > 3 cM were included. IBD pairs summing more than 72 cM or less than 12 cM were excluded [22]

Population	Median sum ROHs (Mb)	Median sum IBD (cM)
Merchero1	19.726	13.587
Merchero2	17.05	14.971
Merchero3	36.906	12.235
Basque	44.348	35.327
Roma	63.198	57.181
Spanish	21.918	15.438
CEU	19.657	15.179
YRI	8.196	14.862

**Fig. 3:**
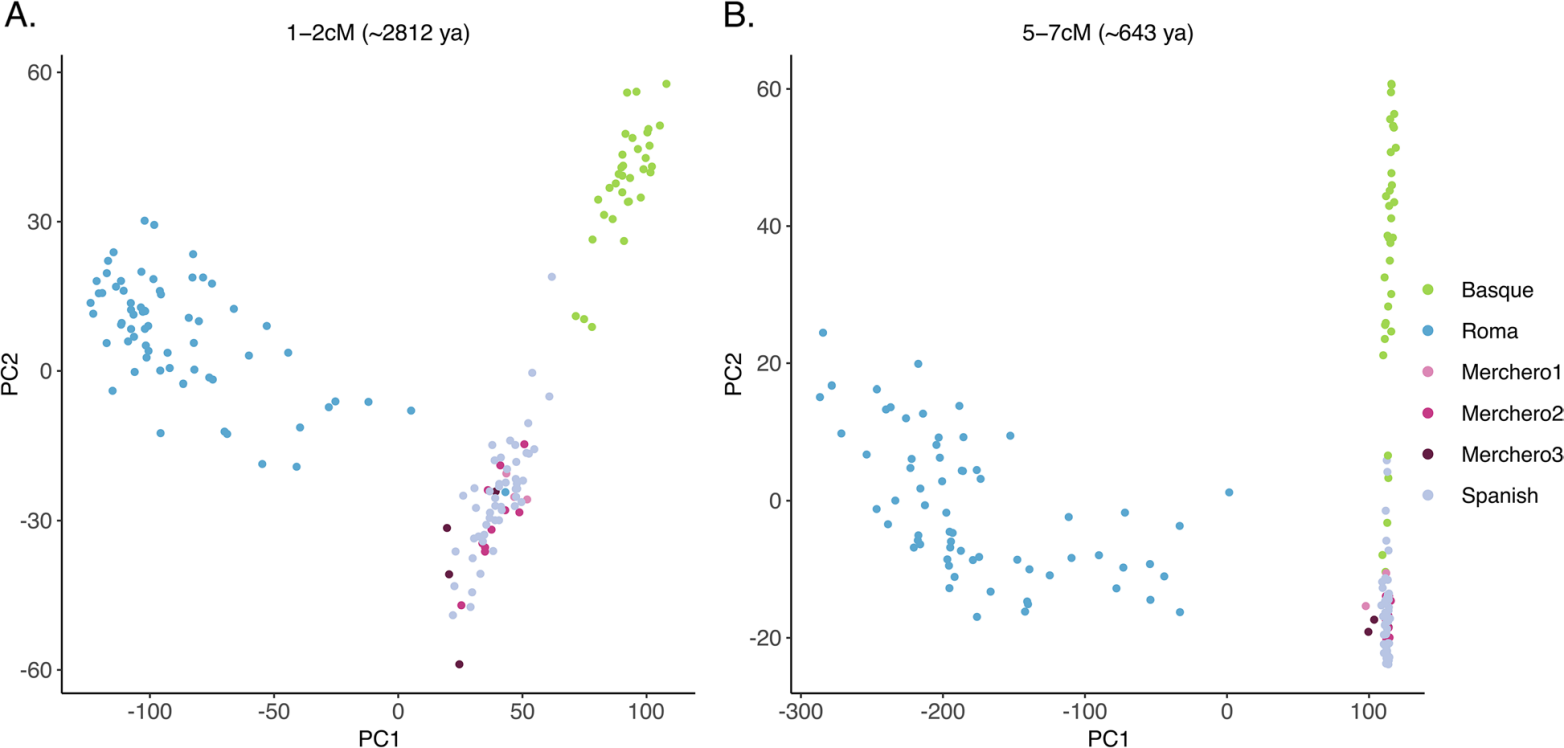
The genomic scenario in the Spanish context over time. The most informative results are shown in a PCA for the IBD length bins of 1–2 cM (**A**) and 5-7 cM (**B**), corresponding to ~ 2812 and ~ 643 years ago, respectively

The global pairwise sharing of IBD segments was explored to test for the genetic relationship among populations. These analyses mirror the results presented above within the Iberian Peninsula context (Fig. S3). Spanish Roma and Basques show large amounts of IBD segments shared internally, in contrast to what is shown in non-Roma Spanish and Mercheros (Fig. S7). Interestingly, the probabilities of sharing IBD between all Merchero clusters and Spanish are similar to the probability of Spanish samples internally sharing IBD segments. This result rejects the hypothesis that Mercheros originated from a founder event from a subset of Spanish individuals. Moreover, the probabilities involving Roma are higher for the Merchero3 cluster, suggesting a genetic closeness between these groups (Table S7). Since the length of the IBD fragments is inversely related to age due to recombination processes, several time depths were explored. Total IBD was decomposed by different length intervals to examine the genetic scenario along the corresponding time periods (Fig. 3). A stable scenario is observed over time regarding short and medium bins of IBD length (< 5 cM), where the Merchero samples fall homogeneously within the Spanish context, whereas Spanish Roma and Basques depict their singular genetic differentiation (Fig. 3A). However, considering long bins (5–7 cM) a slight affinity to Spanish Roma is observed in some Merchero samples, corresponding to a period of time around six centuries ago (Fig. 3B).

These results, in agreement with the previous section, suggest a very recent scenario of genetic inbreeding and substructure in Mercheros, that might be the result of a subtle admixture with an already inbred population such as Roma (Fig. 2B).

We further examined the genetic demographic history of Mercheros focussing on their Ne dynamics. Long-term Ne (200 to 2000 generations ago) was estimated from genome-wide patterns of linkage disequilibrium, showing slightly lower values in Mercheros than in the Spanish general population (Fig. S8A), although sharing a similar trend through time (Fig. S8B). Recent Ne dynamics (0 to 200 generations ago) were next investigated from IBD segments. Mercheros show a Ne decrease contemporaneous to the Black Death (20 generations ago) without recovery, whereas the Spanish group experiences a population reduction around the same age followed by an increase in their Ne (Fig. S9). Although this result might point to a continuous decline in Mercheros Ne, it is important to note that these values are estimated assuming an homogeneous population [23], and as mentioned above, Mercheros present a varying amount of IBD segments, especially in the largest categories. Thus, this result is probably reflecting the Ne trend of some individuals, not the entire population. However, no estimates of the recent Ne can be tested separately for each Merchero group inferred with fineSTRUCTURE due to the low sample size of the genetic clusters.

## Discussion

Several hypotheses have been suggested regarding the origin of Mercheros. However, these hypotheses have not been supported by robust data and they have mostly spread by word of mouth [15]. Our study provides for the first time genetic data from Merchero volunteers and suggests that a common genetic origin with other Spanish groups is the most plausible hypothesis as shown by their genetic affinity and the presence of uniparental lineages commonly found in western European populations (Fig.1, Fig.2, Fig. S1, Fig. S2, Fig. S3 and Fig. S4, Table S1, S4-S5.). Nevertheless, the scarce literature and the lack of other genetic studies focused on the Merchero population challenges the contextualization and interpretation of the analyses and results. Thus, no details can be reported about the specific period and source through which the present Merchero population originated (e.g. landworkers that abandoned the feudalism system during medieval times [15, 17, 18]).

The common origin between Mercheros and Spanish Roma has been suggested due to their cultural similarities, such as a nomadic history and common traditional occupations [15, 17, 18]. Moreover, in spite of being an unclassified language, the *quinqui* exhibits an influence from Spanish and Portuguese Romani (*Caló*) and Basque Romani (*Erromintxela*) [15, 16]. However, our study shows no evidence of a common origin between these groups. Instead, moderate and uneven admixture with Roma is suggested regarding the ancestry proportions inferred in the ADMIXTURE and NNLS analyses (Fig. 1, Fig. 2, Fig. S2, Fig. S3B). No clear admixture events were detected with GLOBETROTTER, supporting that the level of gene flow is too limited to provide robust estimates. Nonetheless, the study of IBD segments shows that those Merchero samples with Roma-related ancestry (Mercheros3; *n* = 4) present a higher probability of randomly sharing segments (Table S7), and the analysis of bins pointed out a very subtle genetic affinity between Mercheros and Spanish Roma in the longest segments of IBDs (Fig. 3). These IBD lengths suggest a contact period which is consistent with the arrival of the Roma people to the Iberian Peninsula, circa 1425 CE [24].

The Merchero population does not represent a genetically isolated and homogenous group within the Iberian Peninsula, which is consistent with previous literature about the social aspects of this population [15, 17, 18]. We show that their origin cannot be explained by a founder effect from the Spanish general population and we describe multiple evidences of intrapopulation substructure. Merchero samples are scattered within the rest of Spanish samples, both in allele-frequency and haplotype-based methods, where different genetic clusters can be identified (Fig. 1 and Fig. 2). These Merchero subgroups have different levels of individual inbreeding: those samples with traceable Roma ancestry display higher number and lengths of ROHs than the rest (Table 1). However, all groups show similar IBD values, lower than those of Roma and Basque populations and comparable with the general Spanish population (Table 1). These results suggest that Mercheros, although being a minority ethnic group and historically persecuted [15, 17, 18], can be defined as a genetically heterogeneous and open population. However, they show a slightly lower genetic diversity compared to the rest of the Spanish population, as shown by the lower Ne values.

## Conclusions

The present study represents the first comprehensive genetic analysis of the Spanish minority ethnic group known as Mercheros. Since ethnicity is influenced by both genetic ancestry and cultural identity, further studies are needed to reconstruct the history of the Merchero population from different scientific disciplines, such as linguistics, anthropology, and genetics. Despite the large amount of publicly available genetic data, there is a considerable gap regarding underrepresented populations and ethnic groups. Overcoming this limitation is pivotal to properly expand the diversity frame of human population genetic studies. This will broaden the knowledge about the complete human population history and the demographic processes that have shaped the genetic variation in fine-scale details, and thus, to improve and facilitate the interpretation of biomedical studies as well [4, 5]. Therefore, we encourage the inclusion of more underrepresented populations and ethnic groups to expand our knowledge about human populations and thus, reduce the overgeneralization, especially regarding the current European ascertainment bias.

## Materials and methods

### Samples, genotyping, and quality control

Twenty five samples were collected from self-reported Merchero volunteers, whose written informed consent was obtained. The parents and grandparents of all volunteers self-identified as Mercheros from several locations in Spain. Moreover, eleven new Spanish Roma samples were collected in order to test for a common origin between Mercheros and Roma, as previously suggested [15]. DNA extraction was performed from saliva samples using a standard protocol, and they were genotyped with the Axiom™ Genome-Wide Human Origins Array (~ 629,443 variants). Genotype calling was performed with the software Axiom™ Analysis Suite 4.0 following the Affymetrix Best Practices Workflow. Twenty samples passed the process with an averaged quality control call rate of 99.7%. Furthermore, 600,225 autosomal single-nucleotide polymorphisms (SNPs) passed the recommended thresholds and were exported to perform the quality control filtering by using PLINK v1.9 software [25]. A filter for more than 10% missing SNPs per individual was applied, resulting in no sample exclusion. Then, SNPs that were missing in more than 5% of the samples, with an extreme deviation from Hardy-Weinberg equilibrium (*p* < 10^− 5^), and a minor allele-frequency (MAF) below 0.05 were filtered out, remaining a total of 599,176 SNPs. Linkage disequilibrium (LD) pruning was performed with a window size of 200 SNPs, a sliding shift of 25 SNPs, and an r^2^ of 0.5 keeping 231,582 SNPs. Three Merchero samples were removed from the dataset due to high level of relatedness (third degree; PI_HAT > 0.125).

To have a reference panel, our dataset was merged with the West Eurasian and North African samples from Lazaridis et al. 2016 study [3]; Utah residents with Northern and Western European ancestry (CEU), Punjabis (PJL), and Yoruba (YRI) from 1000G [1]; and 55 additional Spanish Roma samples [26]. The final dataset includes 1349 samples, 449,160 linked SNPs, and 195,541 unlinked SNPs, for haplotype- and allele-frequency based analyses, respectively.

### Wide- and fine-scale population structure analysis

A PCA was performed using the SmartPCA program from the EIGENSOFT 6.0.1 package [27]. Uniform manifold approximation and projection (UMAP) analysis was run from the first ten PCs with the *umap* method [28] in R [29] with combinations of different “number of neighbors” (2, 5, 10, 20, 50, 100, 200) and “minimum distance” (0.1, 0.25, 0.5, 0.8, 0.99). Model-based individual ancestries were explored with ADMIXTURE v1.3 software [30]. The unsupervised method was run for K ancestral components from 2 to 10 using random seeds in ten independent iterations. Pong v1.4.7 software [31] was used with default parameters to obtain the major modes in the ADMIXTURE results.

In order to better assess population structure and detect fine-scale ancestry patterns, haplotype-based methods were applied. SHAPEIT v2 [32] was first used to perform the phasing of the data, using the HapMap GRCh37 genetic map [2] and the 1000G dataset as a reference panel [1]. The data was aligned to the reference and the mismatched SNPs were removed, then the proper phase inference was performed. Secondly, ChromoPainter v2 was used to infer the total length and count of haplotype fragments shared between individuals [33]. ChromoPainter was first run to estimate the global mutation probability and the switch rate parameters by running 15 iterations of the expected-maximization (EM) algorithm over chromosomes 1, 4, 17, and 20. These inferred values were averaged across the four chromosomes and samples. The parameters were used to run ChromoPainter for all chromosomes and individuals in order to obtain the final coancestry matrices of count and length sharing. Next, matrices across all chromosomes were summed by using ChromoCombine to obtain the copying profile for each individual and the C parameter required for running fineSTRUCTURE [33]. FineSTRUCTURE v2.1.0 was launched in order to cluster the data obtained from ChromoPainter into homogeneous genetic groups. The analysis included 2 million MCMC iterations, with 1 million burn-in iterations and sampling values from the posterior probability every 10,000 iterations. Three different runs of the analysis were performed for three different seeds to check the consistency of the analysis. An analysis of molecular variance (AMOVA) was performed with the *poppr* v2.1.0 R package [34] to analyse the homogeneity of these groups. *P*-values were obtained through the empirical distribution under the null hypothesis from 1000 permutations with the *ade4* v2.0.1 R package [35]. After grouping all individuals into genetically homogeneous clusters, ChromoPainter was run for these clusters and ancestry profiles were estimated using the NNLS method implemented in *nnls* v1.4 R package [36]. GLOBETROTTER [37] was used to test for admixture events. Following the recommended procedure, null.ind parameter was set to 1 in order to test for plausible admixture events using 100 bootstrap resamples. Then, null.ind parameter was set to 0 to characterize the event and estimate the admixture sources, proportions and dates. Finally, confidence intervals for the dates were inferred through 100 bootstrap iterations, considering one- and two-date admixture models.

### Inbreeding estimation: ROHs and IBD segments

ROHs were identified using PLINK v1.9b software [25] with the following non-default parameters: maximum gap between SNPs of 100 kb and a minimum threshold of 500 kb and 50 SNPs. This analysis was performed using a set of reference populations to enable an informative comparison: Spanish as representative of the general Iberian Peninsula context; Basque and Roma as examples of ancient and recent inbreeding, respectively [11, 13, 21]; CEU and YRI which represent the genetic diversity with and without the Out-of-Africa bottleneck, respectively. We performed Wilcoxon tests to assess whether the sum of ROH lengths between each pair of populations was statistically significant.

IBD segments were identified using IBDSeq (version r1206) software [38] with default parameters. HapMap GRCh37 genetic map [2] was used to convert base pairs to genetic positions in cM. To construct an IBD heatmap, we first excluded IBD segments shorter than 3 cM [39], we summed the IBD pairwise lengths between individuals and removed those individual pairs whose IBD sharing was lower than 12 cM and higher than 72 cM [39]. We then constructed a heatmap with the *ggplot2* R package [40] showing theIBD sharing between pairs of individuals. Wilcoxon tests were performed to statistically assess the significance of the differences between “within-population” IBD sharing lengths of each pair of populations. We next performed PCA with *prcomp* function in R base [29] with the sum of IBD pairwise lengths computed separately using IBD segments with 1–2 cM and 5–7 cM to gain insights on the temporal population structure. To approximate the expected age of the IBD segments in these two bins (1-2 cM; 5-7 cM), we applied the formula described previously by Byrne et al. [41], assuming a generation time of 25 years. Although the length of IBD segments represents wide time distributions, these expected values are point estimates that can be used as a reference. In addition, we computed the probability that an individual selected at random from one population shares an IBD pairwise length greater than 7 cM with an individual selected at random from another population, after excluding IBD segments lower than 3 cM, as previously described [42].

### Effective population size (ne) inference

To estimate the Ne trajectories (0 to 200 generations ago), we used IBDNe (version 23Apr20.ae9) software [23] with default parameters with the IBD segments identified with IBDSeq [38]. In addition, long-term Ne values (200 to 2000 generations ago) were estimated using the NeON v1.0 R package [43] with default parameters.

### Uniparental markers analyses

Mitochondrial DNA (mtDNA) was PCR-amplified in four fragments using 4 pairs of primers under identical conditions [44]. Genetic libraries were prepared and sequencing was performed by following the *Illumina mtDNA Genome* [45] and *Illumina MiSeq* guidelines [46], respectively. Mapping was performed according to GATK best practices [47] and individual haplogroups were assigned with HaploGrep [48]. Only twenty three out of twenty five mtDNA sequences passed the following quality thresholds: a minimum of 15X of coverage in all four amplified regions and a HaploGrep quality score of at least 80% [49] (Table S1).

Seventeen STRs present in the commercially available AmpFLSTR Yfiler PCR Amplification Kit (Thermo Fisher Scientific) were genotyped following manufacturer’s recommendations. Y-chromosome haplogroups were predicted from YSTRs using Whit Atheys’ haplogroup predictor (http://www.hprg.com/hapest5/) [50].

## Supplementary Information

**Additional file 1.** Supplementary Information file. Additional Figures (Fig. S1-S9) and Tables (Table S1- S7) with their references.

## Data Availability

Genome-wide array data and whole mtDNA sequences have been deposited at the European Genome-phenome Archive (EGA), under accession number EGAS00001005360.
