# Supplementary material for: The genetic scenario of Mercheros: an under-represented group within the Iberian Peninsula
Source: BMC Genomics. 2021 Dec 15;22:897. doi: 10.1186/s12864-021-08203-y (PMC8672588; doi:10.1186/s12864-021-08203-y)
Supplement: Supplementary file 1 — Additional file 1. Supplementary Information file. Additional Figures (Fig. S1-S9) and Tables (Table S1- S7) with their references. [file 12864_2021_8203_MOESM1_ESM.docx]

**Supplementary Information**

**The genetic scenario of Mercheros: an under-represented group within the Iberian Peninsula**

André Flores-Bello^1,*^, Neus Font-Porterias^1,*^, Julen Aizpurua-Iraola^1^, Sara Duarri-Redondo^1^, David Comas^1^

^1^Departament de Ciències de la Salut i de la Vida, Institut de Biologia Evolutiva (CSIC-UPF), Universitat Pompeu Fabra, Barcelona, 08003, Spain

^*^These authors equally contributed to this work

Corresponding author: [david.comas@upf.edu](mailto:david.comas@upf.edu)

**Supplementary Figures**

**
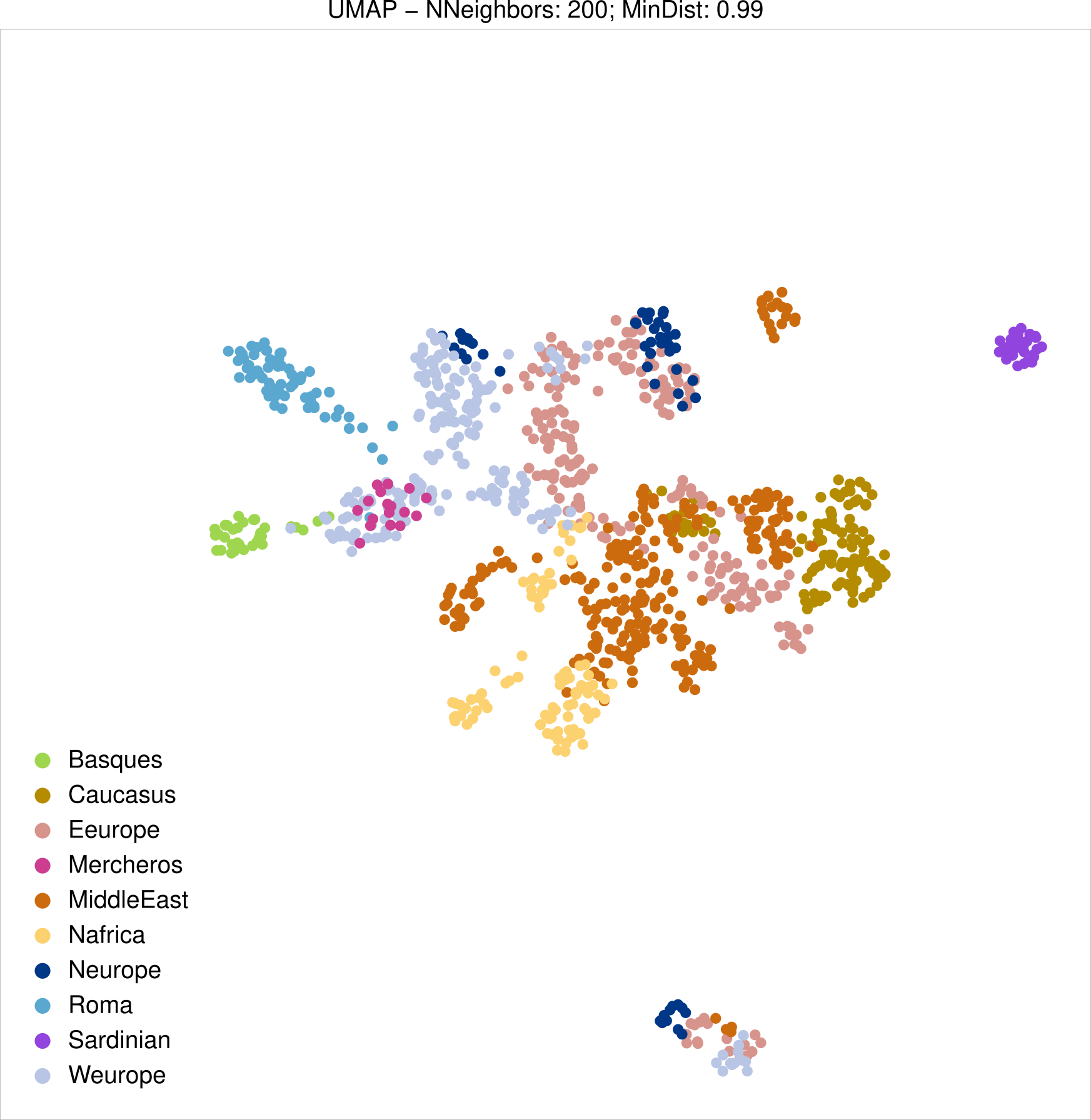
**

**Figure S1. UMAP projection of the first ten PCs including West Eurasian and North African individuals (number of neighbours = 200; minimum distance = 0.99).** Eeurope = Eastern Europe; Nafrica = North Africa; Neurope = Northern Europe; Weurope = Western Europe.


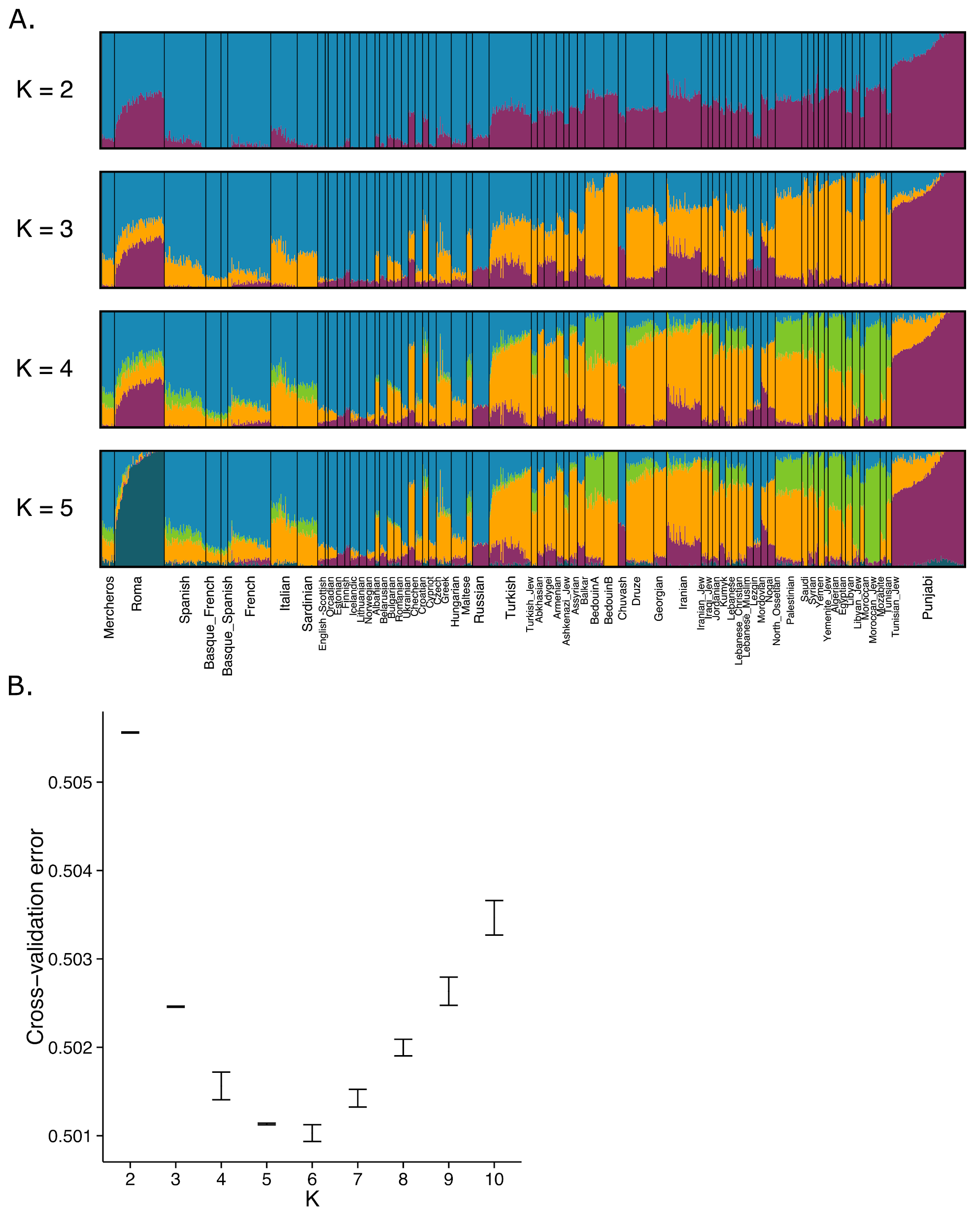


**Figure S2. ADMIXTURE analysis in a wide genetic context.** Results from K = 2 to K = 5 including the whole dataset (**A**) and the cross-validation error values for each K (**B**) are shown. Result for K=6, which is the one with the lowest cross validation error, is shown in Figure 1B.


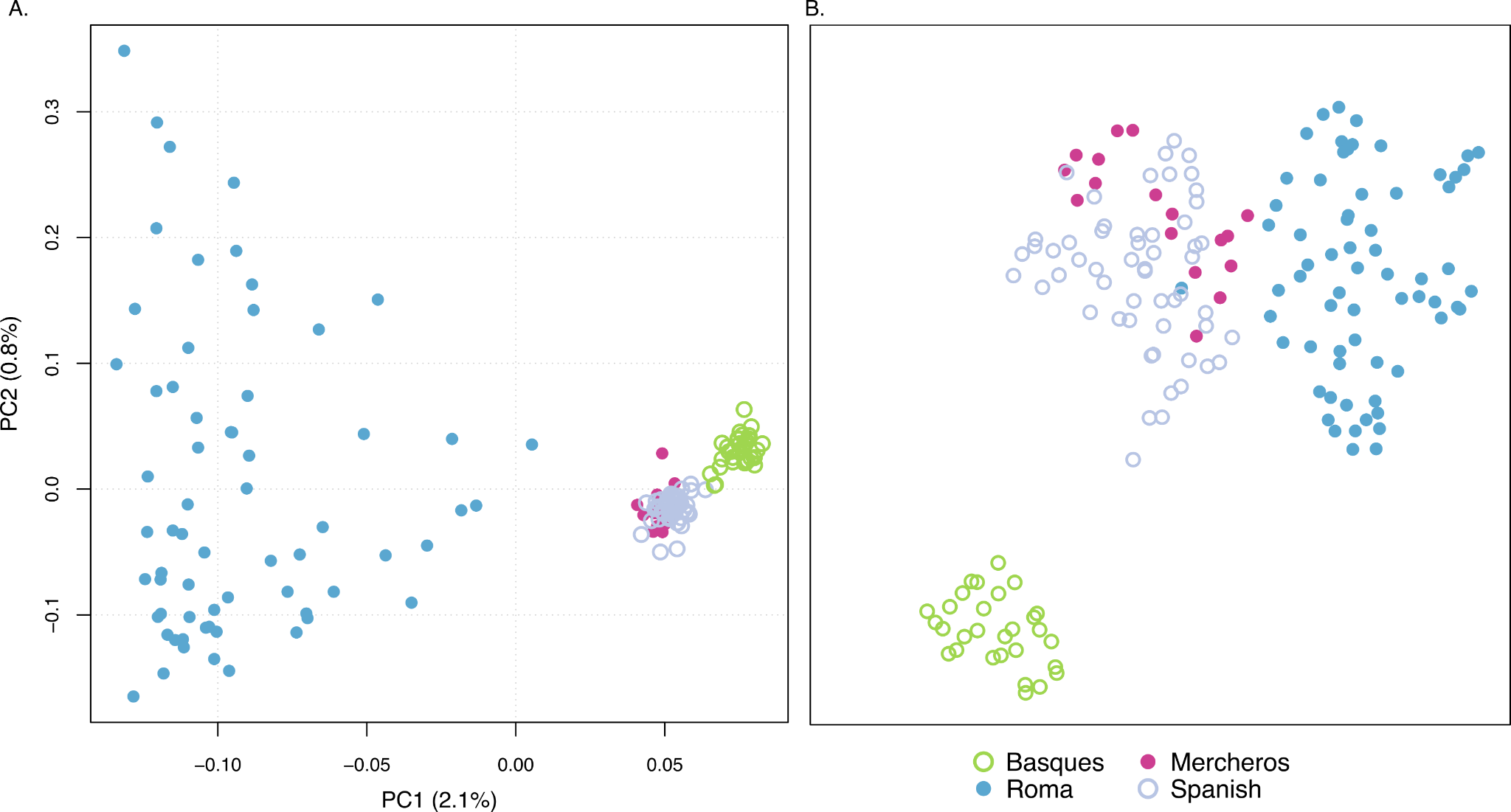


**Figure S3. The genetic scenario in the Spanish context.** PCA analysis **(A)**, and UMAP projection (number of neighbours = 10; minimum distance = 0.5) **(B)**, including individuals from the Iberian Peninsula (considering Basques, Spanish Romani, Mercheros, and Spanish general population). Color legend is the same for both panels.


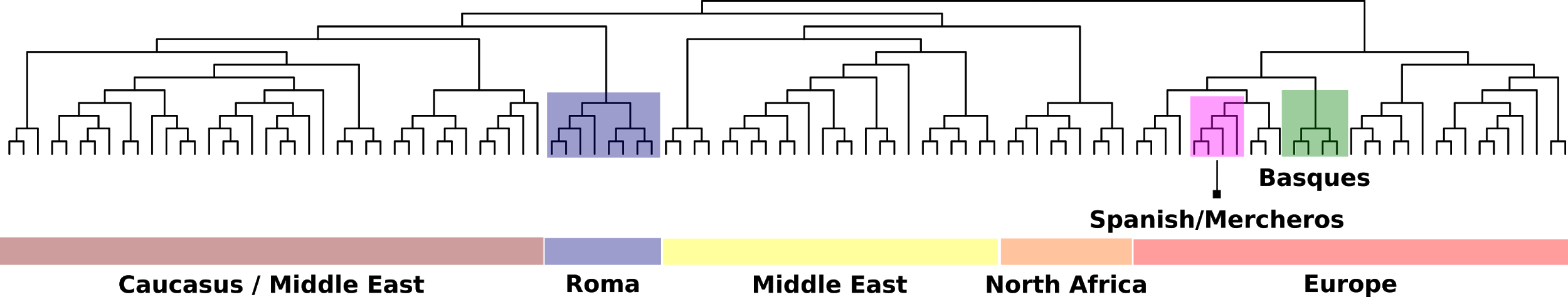


**Figure S4. Full fineSTRUCTURE dendrogram.** General branches are highlighted with colored bars. Clusters composed by geographic Spanish groups are colored in the dendrogram. Purple, Spanish Roma; Pink, Mercheros and Spanish non-Roma/non-Mercheros; Green, Spanish Basques. The specific Spanish/Merchero branch is shown in Figure 2.


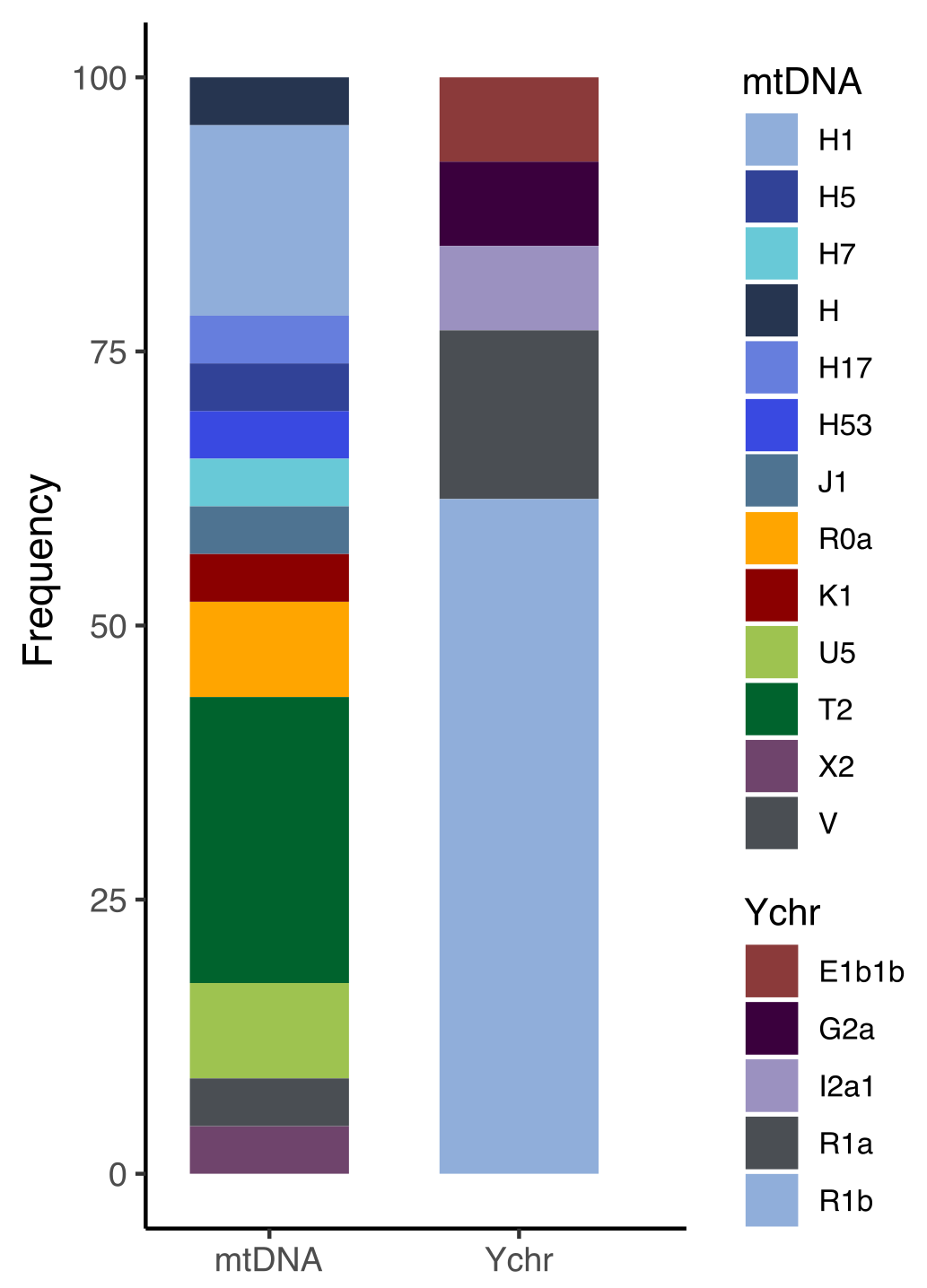


**Figure S5. Haplogroup frequencies of the mtDNA and Ychr Merchero samples.**


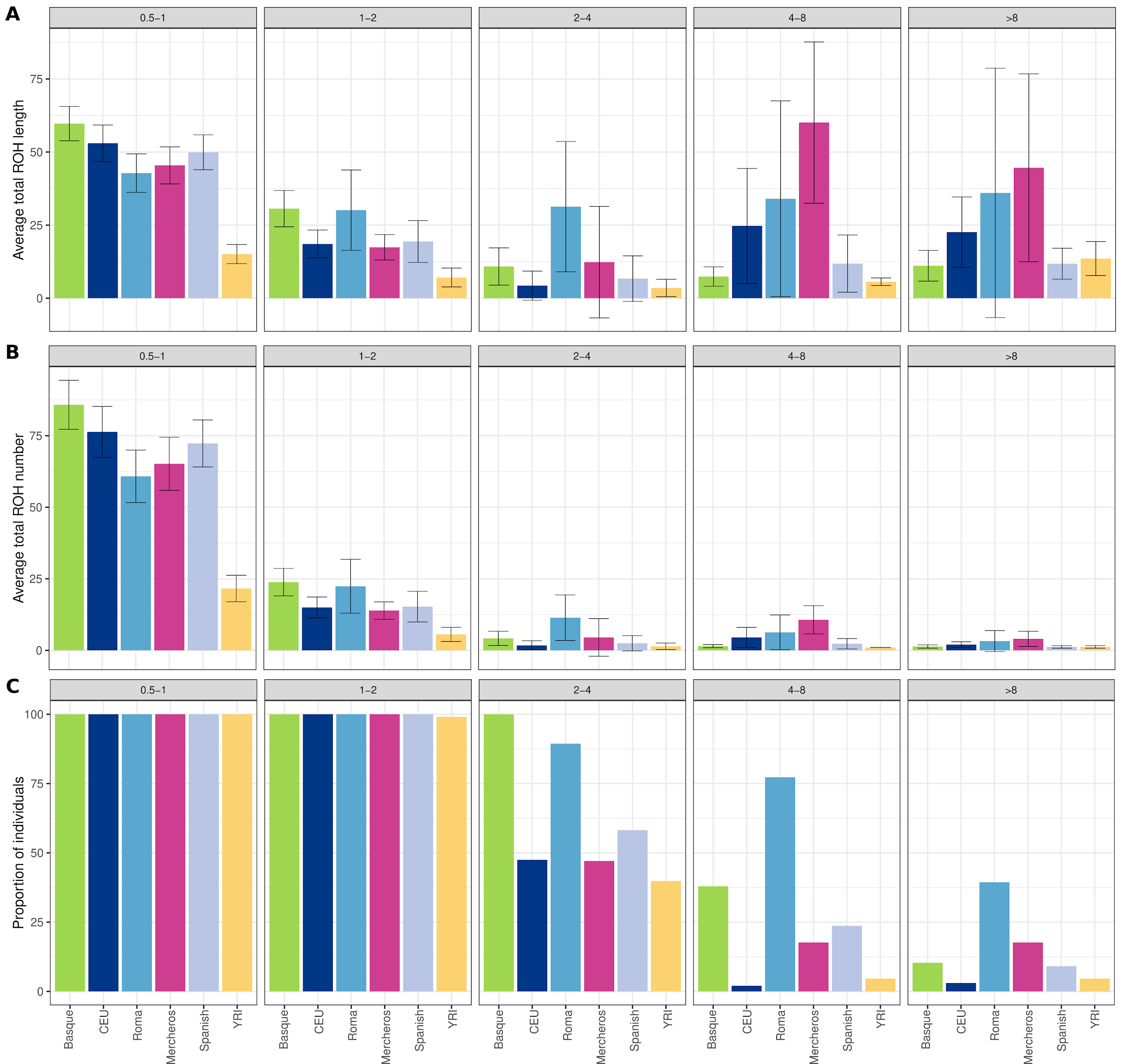


**Figure S6. ROH analysis across different length categories.** **A**. Barplot with the total length of ROH. **B**. Barplot with the number of ROHs. **C**. Proportion of individuals represented in each ROH category. Mb, Megabases.

**
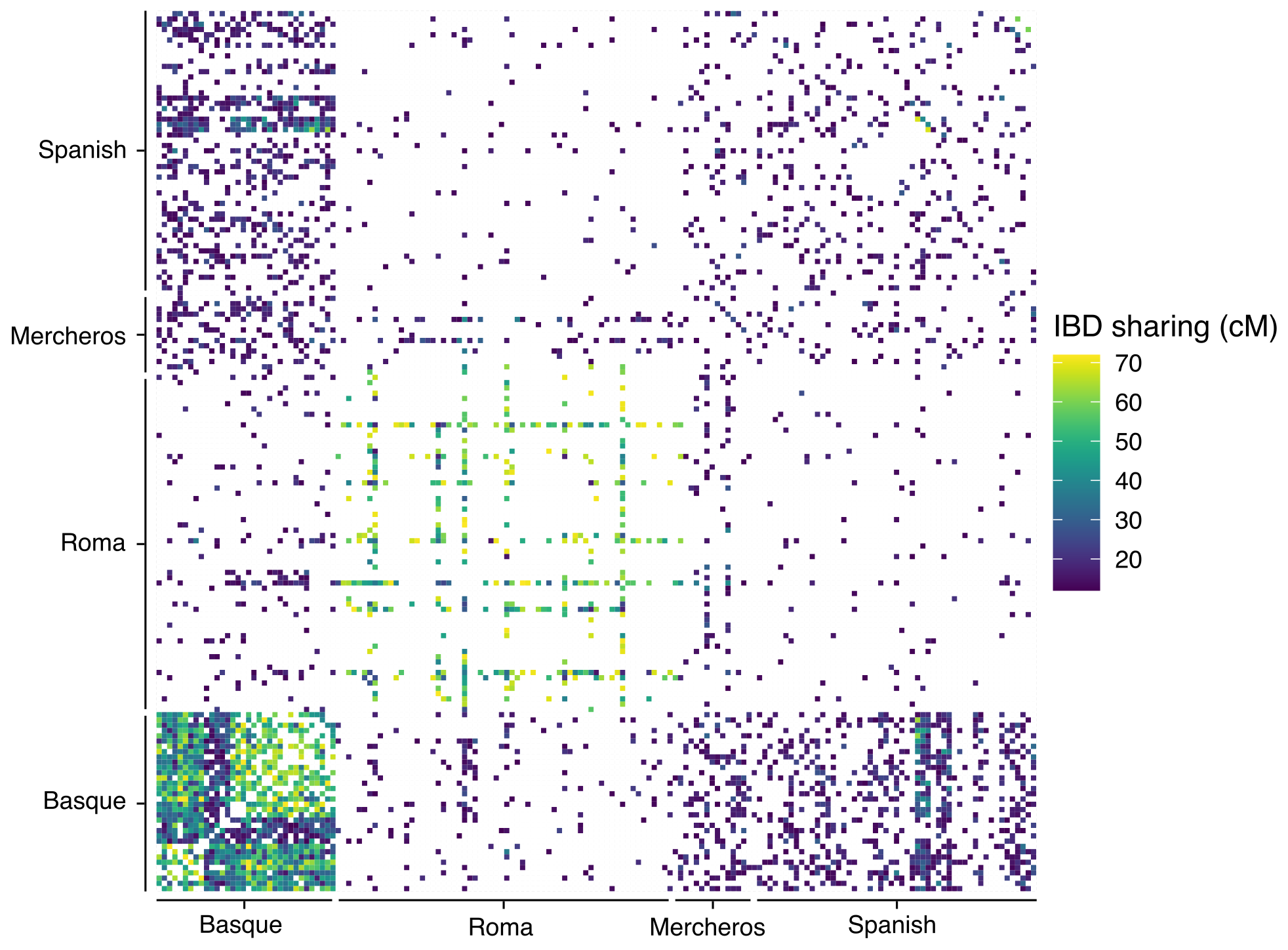
**

**Figure S7. IBD sharing heatmap.** The total length (cM; centiMorgans) of IBD segments shared between samples was used.


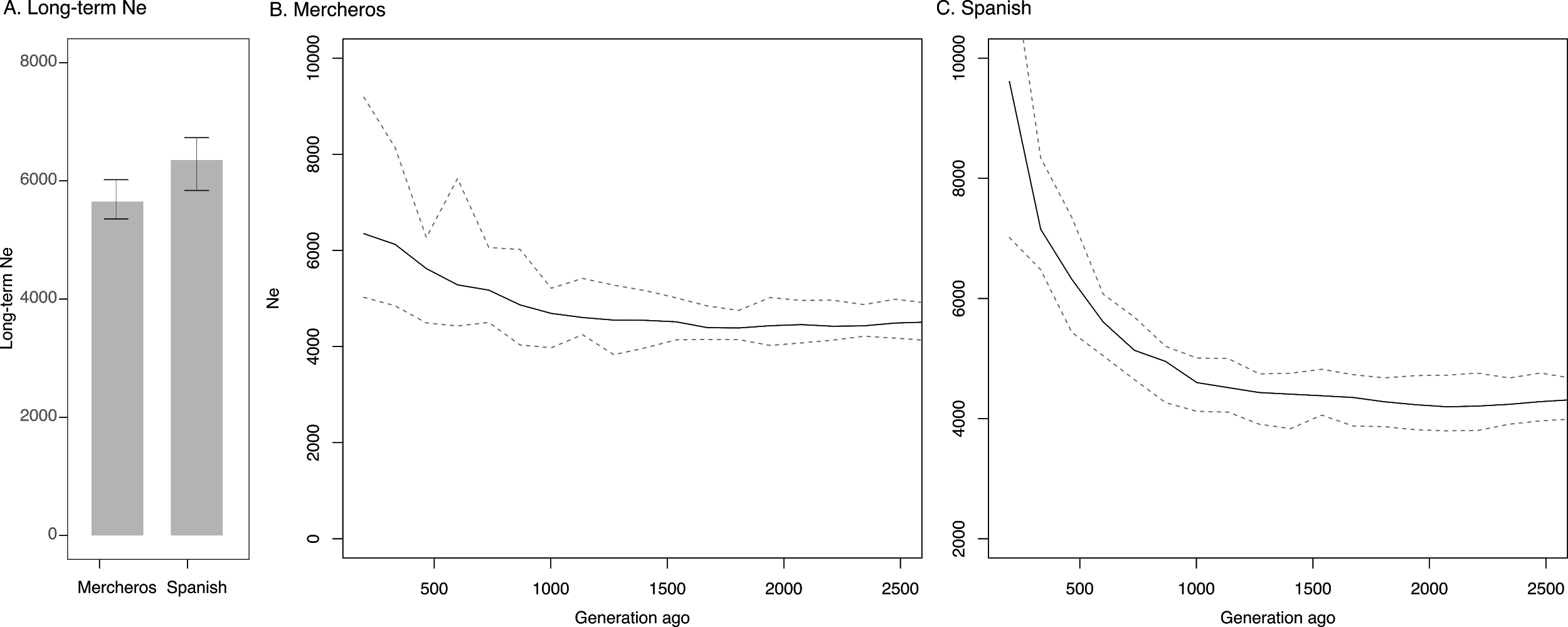


**Figure S8**. **Ne estimates from 200 to 2500 generations ago using NeON** [(1)](https://www.zotero.org/google-docs/?6zQUou). **A.** Long-term Ne values. **B-C**. Ne trend across time for the Merchero and Spanish populations.

**
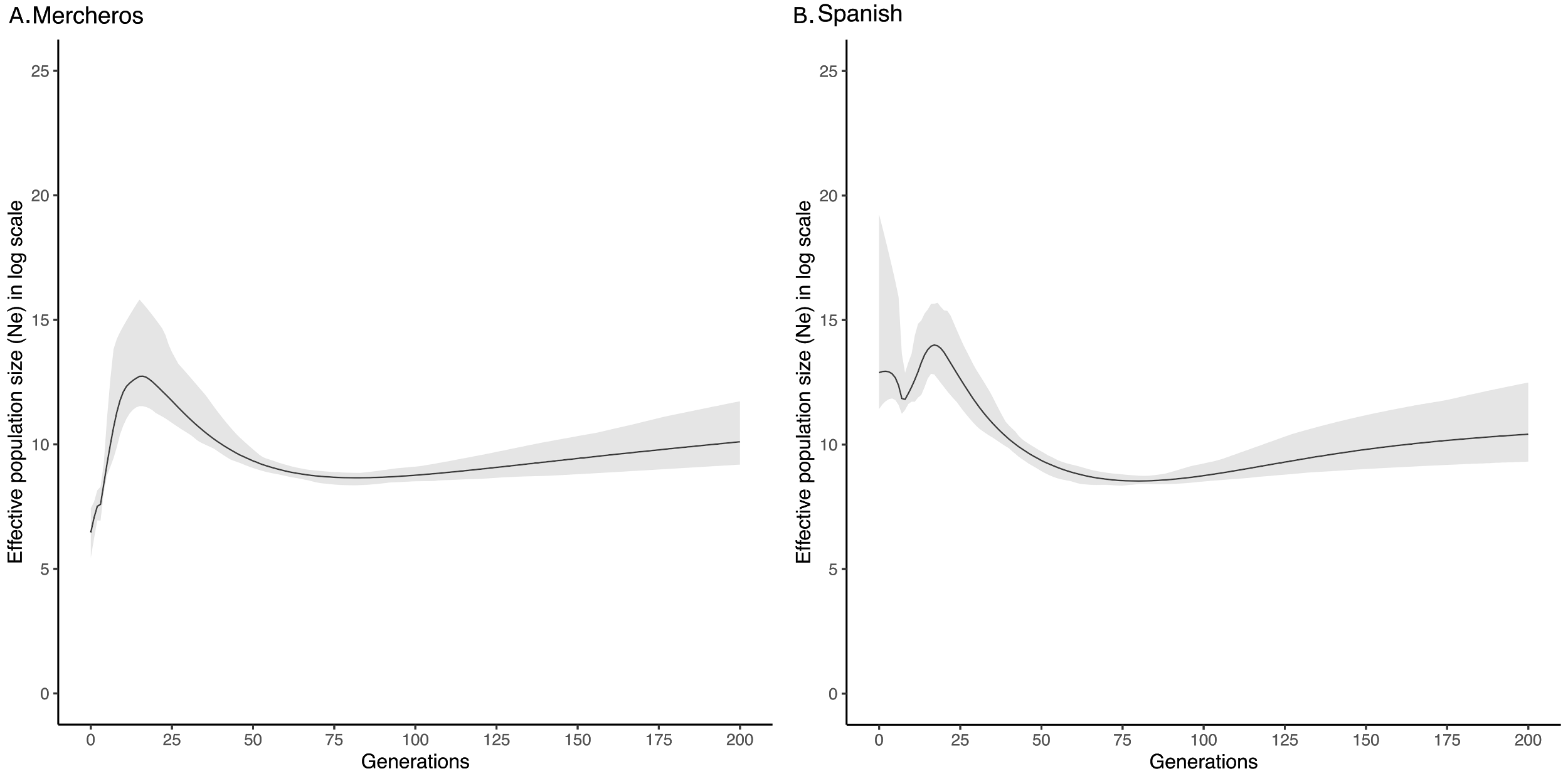
**

**Figure S9. Ne trend estimates from 0 to 200 generations ago using IBDNe**[(2)](https://www.zotero.org/google-docs/?L1nMI9)**.** The analysis was performed for the Merchero (**A**) and Spanish (**B**) populations.

**Supplementary Tables**


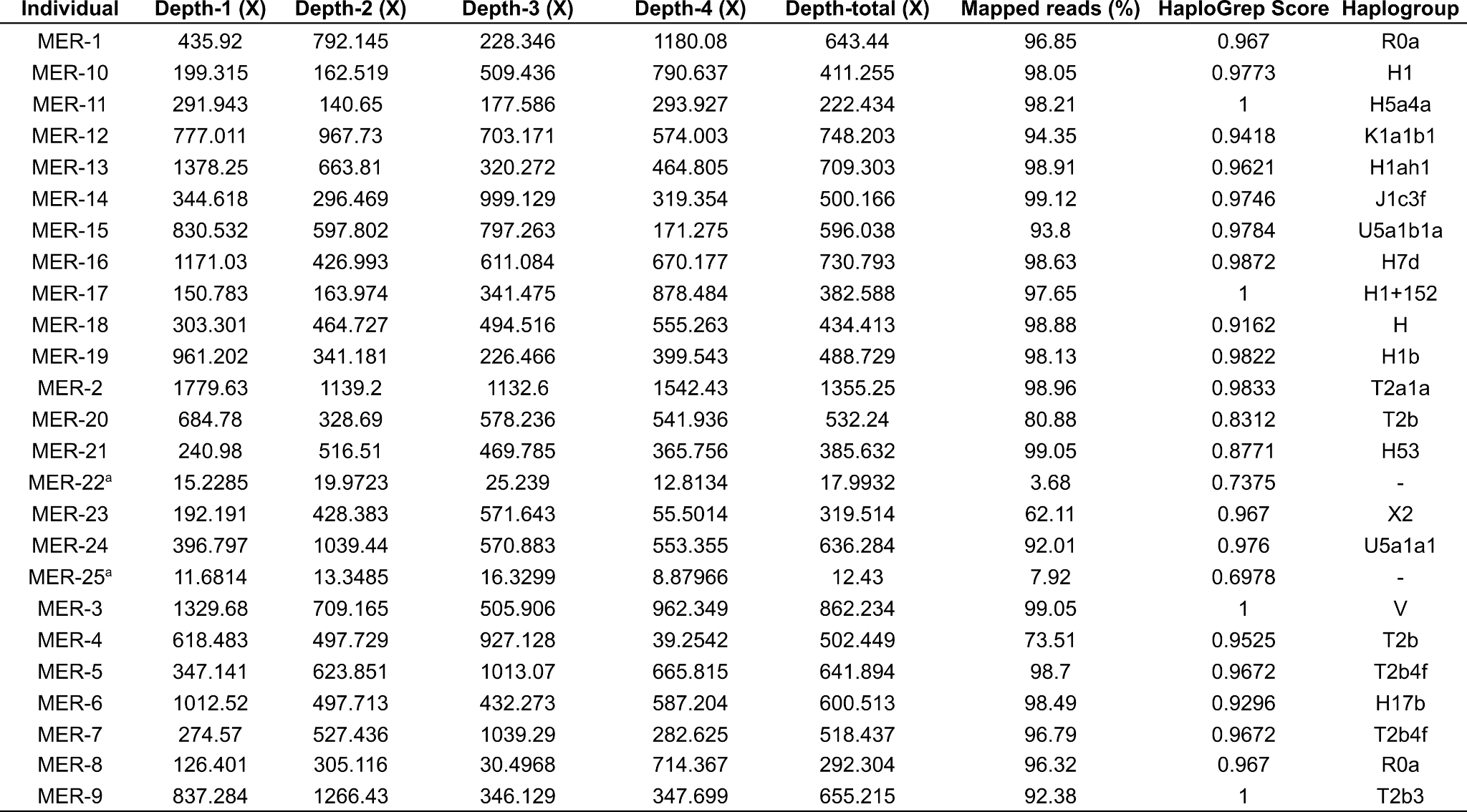


**Table S1. Quality metrics of the mtDNA sequences and haplogroups inferred from HaploGrep** [(3)](https://www.zotero.org/google-docs/?S4j3MK)**.** For each individual sequence, the mean depth of coverage for each of the four amplified fragments, the total mean depth, the proportion of mapped reads, the HaploGrep score, and estimated haplogroups are shown. ^a^Excluded sequences due to low quality (See materials and methods for further details).


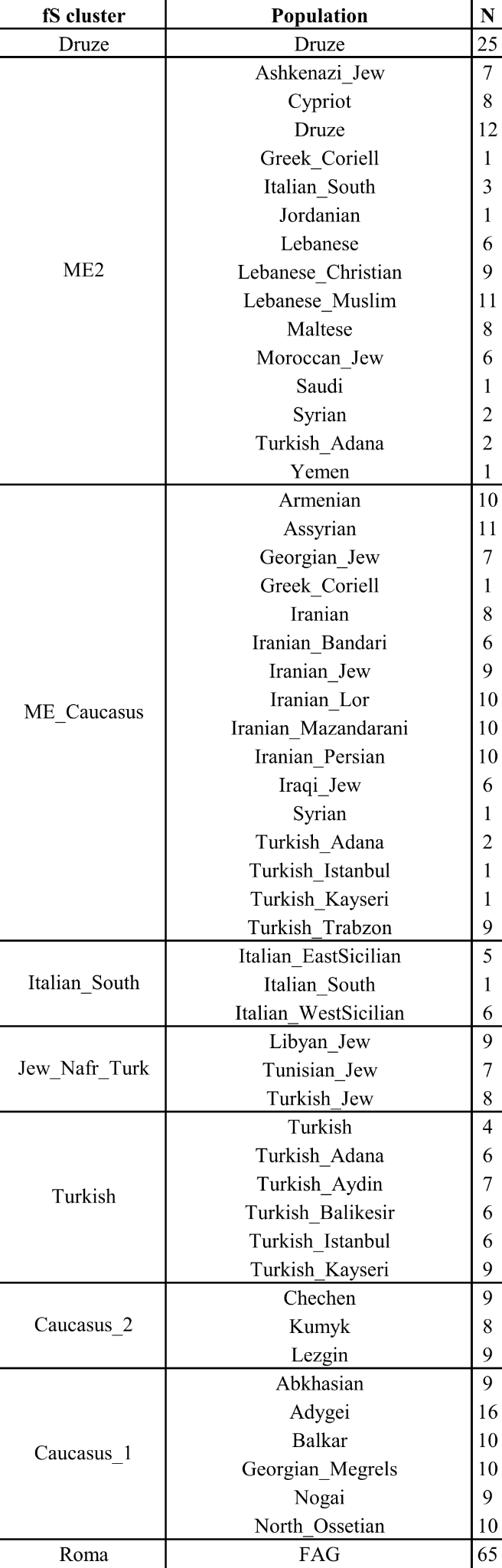

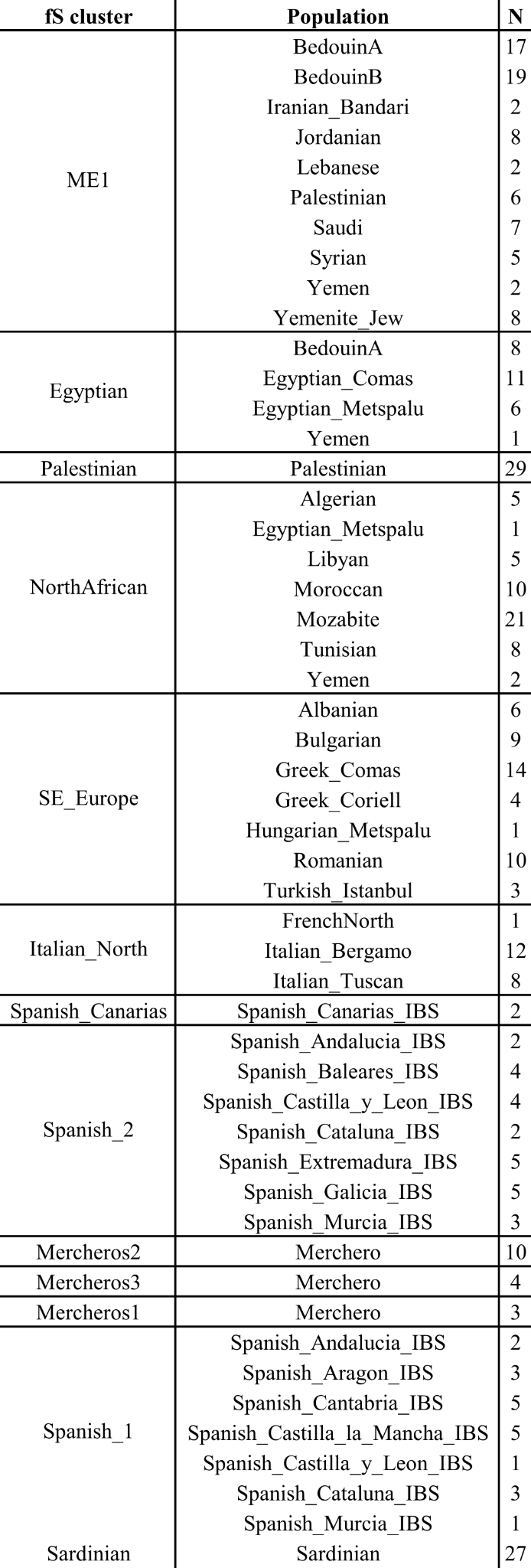

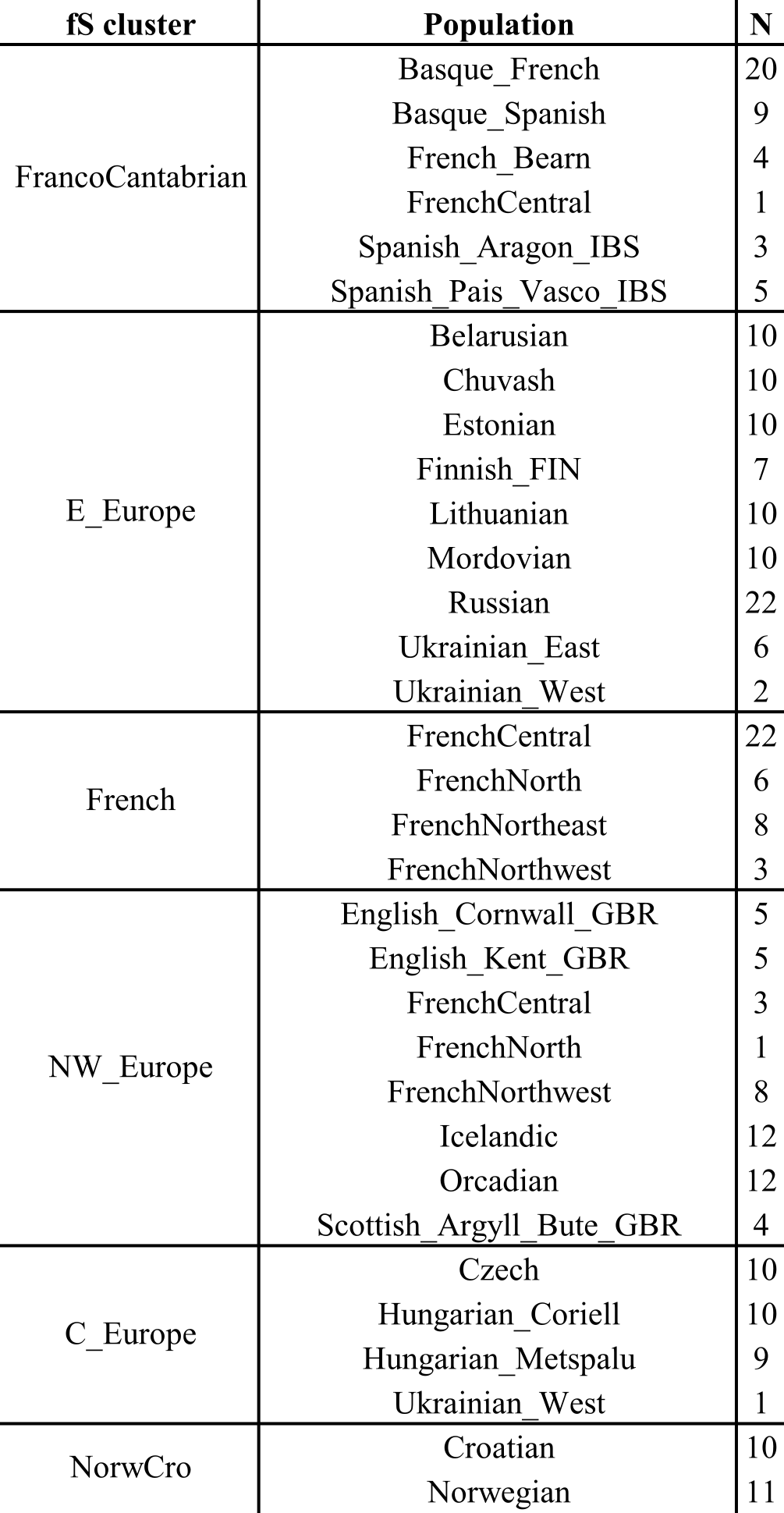


**Table S2. Composition of the clusters obtained from the fineSTRUCTURE dendrogram.** The first column shows the name of the clusters considered for the second step of ChromoPainter, NNLS and GLOBETROTTER analyses. The second column indicates the label of the populations within each cluster. The last column presents the number of individuals from each population included within each cluster.

|  | Merchero1 – Spanish | | Merchero2 – Spanish | | Merchero3 – Spanish | |
| --- | --- | --- | --- | --- | --- | --- |
|  | Explained variation | P-value | Explained variation | P-value | Explained variation | P-value |
| Variation between groups | 0.273 | 0.007 | 0.149 | 0.001 | 1.003 | 0.001 |
| Variation within group | 99.727 | - | 99.851 | - | 98.997 | - |

**Table S3. Analysis of Molecular Variance. The analysis was performed in order to explore the genetic heterogeneity of Merchero clusters.** The variation explained is higher when analyzing the Merchero3 cluster, while Merchero1 and Merchero2 clusters show lower levels of heterogeneity.

**
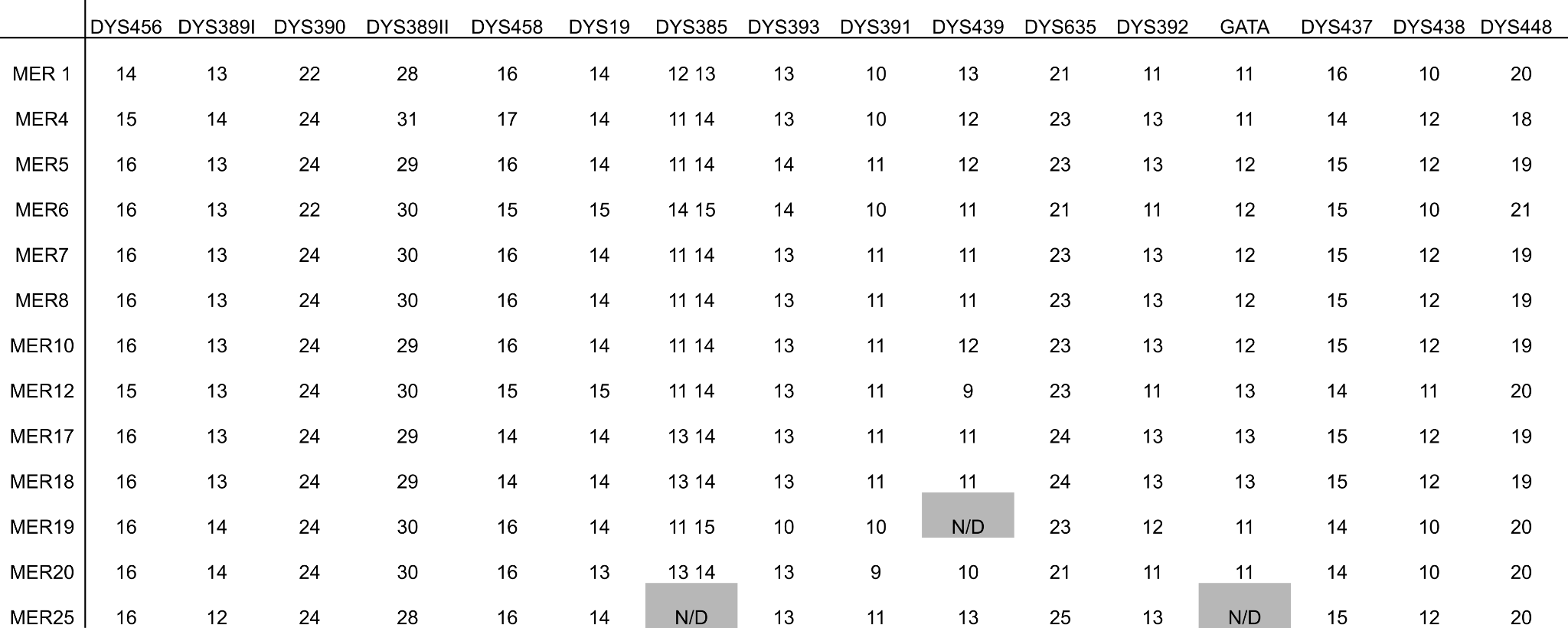
**

**Table S4. Number of copies for each Ychr STR in the Merchero males samples.** N/D, not determined.

**
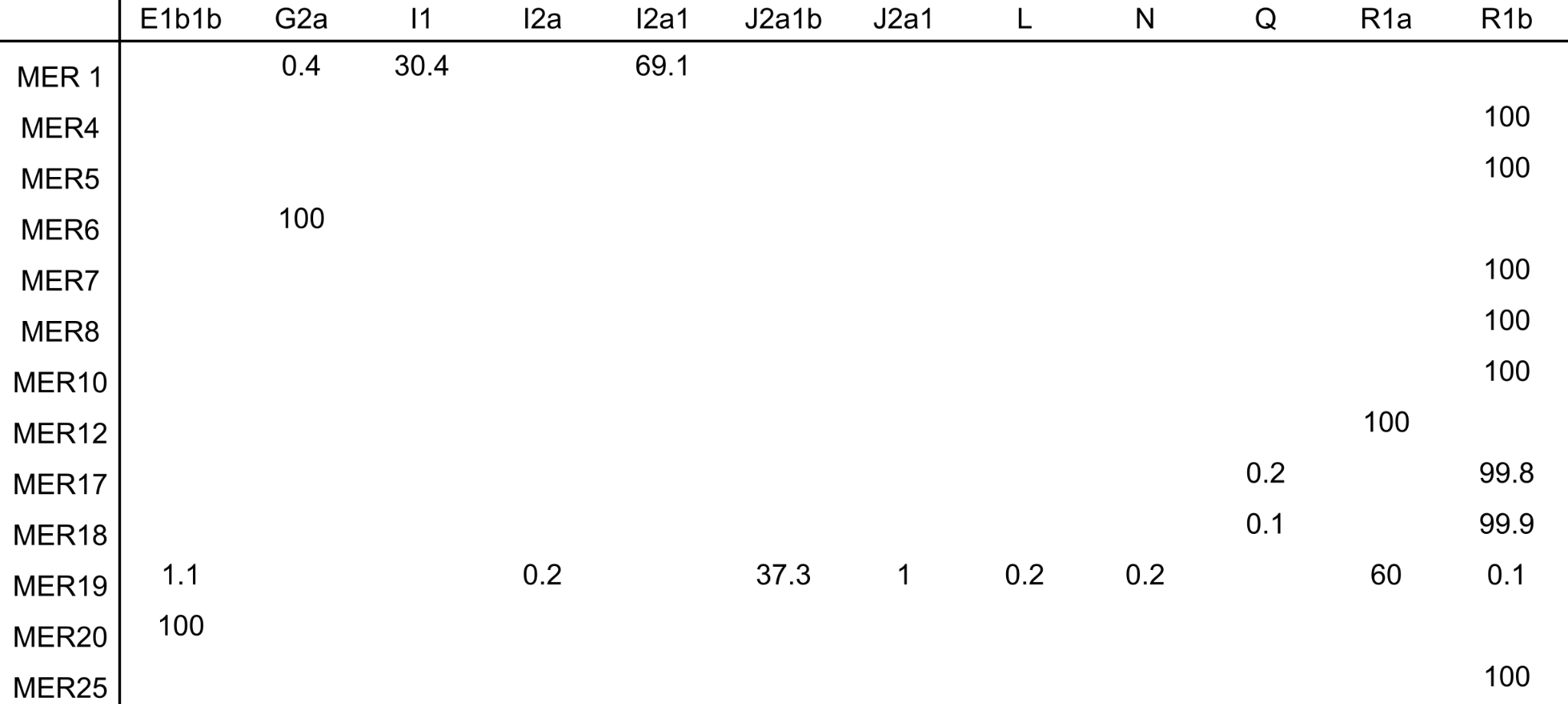
**

**Table S5. Prediction probability (%) of Y-chromosome haplogroup from Y-STR by using hprg (**[**http://www.hprg.com/hapest5/**](http://www.hprg.com/hapest5/)**)** [(4)](https://www.zotero.org/google-docs/?4BeY8q).


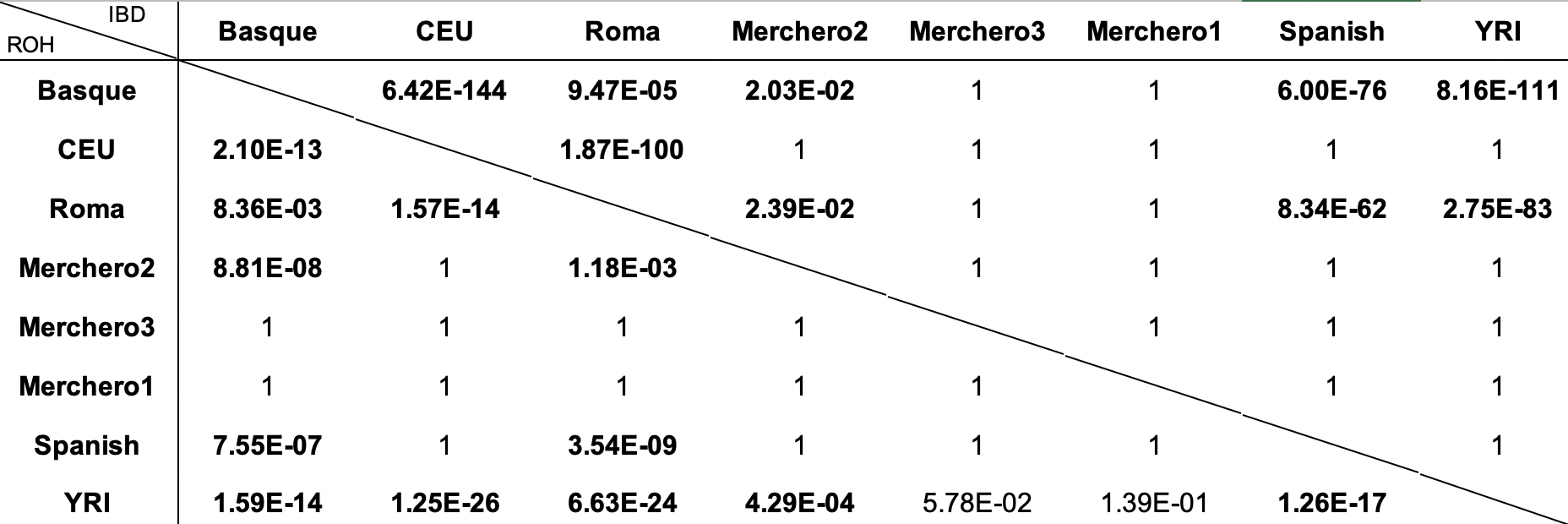


**Table S6. p-values resulting from Wilcoxon tests comparing each pair of populations and the sum of ROH length (lower-left triangle) or IBD sharing lengths (upper-right triangle).** P-values are adjusted for multiple testing with Bonferroni correction. p<0.05 are shown in bold.

|  | **Roma** | **Spanish** |
| --- | --- | --- |
| **Roma** | 0.972 | 0.146 |
| **Merchero1** | 0.288 | 0.427 |
| **Merchero2** | 0.138 | 0.350 |
| **Merchero3** | **0.364** | 0.310 |
| **Spanish** | 0.146 | 0.347 |

**Table S7. Probability that an individual selected at random from a population shares an IBD length higher than 7 cM with an individual selected at random from the other population.** Only IBD segments larger than 3 cM are included [(5)](https://www.zotero.org/google-docs/?efb0gY).

**REFERENCES**

[1. Mezzavilla M, Ghirotto S. Neon: An R Package to Estimate Human Effective Population Size and Divergence Time from Patterns of Linkage Disequilibrium between SNPS. J Comput Sci Syst Biol. 2015;8.](https://www.zotero.org/google-docs/?yDqNQ8)

[2. Browning SR, Browning BL. Accurate Non-parametric Estimation of Recent Effective Population Size from Segments of Identity by Descent. Am J Hum Genet. 2015;97(3):404-18.](https://www.zotero.org/google-docs/?yDqNQ8)

[3. Weissensteiner H, Pacher D, Kloss-Brandstätter A, Forer L, Specht G, Bandelt H-J, et al. HaploGrep 2: mitochondrial haplogroup classification in the era of high-throughput sequencing. Nucleic Acids Res. 2016;44(W1):W58-63.](https://www.zotero.org/google-docs/?yDqNQ8)

[4. Athey TW. Haplogroup Prediction from Y-STR Values Using a Bayesian-Allele- Frequency Approach. J Genet Geneal. 2006;2:34-9.](https://www.zotero.org/google-docs/?yDqNQ8)

[5. Ioannidis AG, Blanco-Portillo J, Sandoval K, Hagelberg E, Miquel-Poblete JF, Moreno-Mayar JV, et al. Native American gene flow into Polynesia predating Easter Island settlement. Nature. 2020;583(7817):572-7.](https://www.zotero.org/google-docs/?yDqNQ8)
